# Structure preserving t-SNE of matrix framed data

**DOI:** 10.1016/j.csbj.2025.04.019

**Published:** 2025-04-16

**Authors:** Soohyun Ahn, Johan Lim, Wei Jiang, Sungim Lee, Xinlei Wang

**Affiliations:** a Department of Mathematics, Ajou University, Suwon, Gyeonggi, Korea; b Department of Statistics, Seoul National University, Seoul, Korea; c Department of Statistics and Data Science, Dankook University, Yongin, Gyeonggi, Korea; d Department of Mathematics, University of Texas at Arlington, Arlington, TX, USA; e Division of Data Science, College of Science, University of Texas at Arlington, Arlington, TX, USA

**Keywords:** Bi-clustering, Dimension reduction, Exergame data, Matrix t-SNE, Microarray gene expression data

## Abstract

Across various fields, we can align data elements into a matrix frame with both row and column indices, forming what we refer to as matrix-framed data. These elements can take various forms, such as scalars, vectors, time series, matrices, or arrays. Existing data visualization methods aim to represent data elements of different groups without considering the underlying two-dimensional structure present in matrix-framed data. To address this limitation, we introduce a novel visualization method called Matrix t-SNE, designed to effectively embed matrix elements into a low-dimensional Euclidean space while preserving both row-wise and column-wise group structures. Our approach extends the classical t-SNE algorithm to accommodate matrix-framed data, providing a detailed algorithmic framework for embedding such data into low-dimensional representations. To demonstrate the effectiveness of Matrix t-SNE, we apply it to three real-world datasets: exergame, gene expression, and temperature. Our results show that Matrix t-SNE achieves more effective separation of elements according to latent row-wise and column-wise group structures compared to the classical t-SNE.

## Graphical abstract

**Figure:**
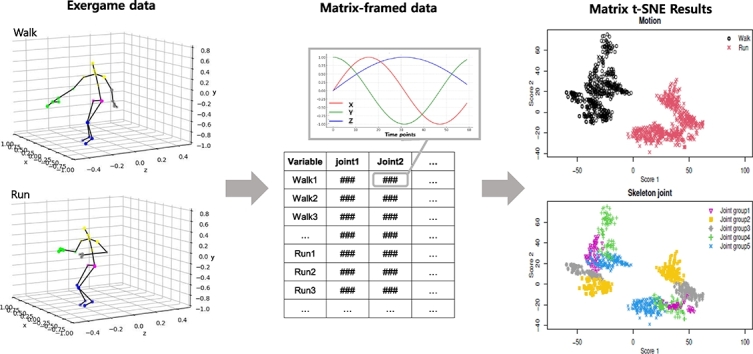

## 1 Introduction

Across various fields, we can effectively organize data elements into a matrix frame, featuring both row and column indices. For instance, in public health research, Park et al. [1] demonstrated the utility of structuring longitudinal blood pressure data as matrices, where rows correspond to systolic and diastolic measurements, and columns represent distinct time points. Similarly, in genomics studies, Madeira and Oliveira [2] analyzed gene expression data that were structured as matrices, with genes represented by rows and conditions or time points represented by columns. Furthermore, in the realm of economics, Tsay [3] organized employment statistics across multiple states into a matrix frame, with state information listed along the rows and employment-related variables along the columns.

We refer to this type of data with elements aligning into a matrix frame as matrix-framed data, which retains a two-dimensional structure that preserves the structural information of the individual elements. Within this type of data, the elements can take various forms, such as scalars, vectors, matrices, or (multivariate) time series. Furthermore, these matrix-framed data sets are characterized by two indexing indicators, which define the respective rows and columns. It is essential to note that not all structured datasets with a matrix representation benefit from preserving row-wise and column-wise group structures. In some cases, the structural information does not inherently exist, making it unnecessary to retain such relationships. For instance, image datasets like MNIST can be represented as matrices of pixels, but they typically do not possess inherent row-wise or column-wise dependencies that require preservation.

To illustrate the importance of preserving matrix structures in certain applications, we examine three matrix-framed datasets that exhibit underlying structural relationships: exergame data (Section 3.1), gene expression data (Section 3.2), and temperature data (Section 3.3). In the exergame dataset, the row index corresponds to individual IDs and the column index stands for joints, where the individuals are grouped by activities (e.g., walking or running) and the joints are grouped by locations. In the gene expression dataset, the row index again represents individual IDs and the column index represents genes, where the individuals are grouped by breast cancer mutation types (either 1 or 2), and the genes are grouped based on similarities. In the temperature dataset, the rows corresponds to different regions, while the columns represent years, capturing both spatial and temporal structures of temperature variations. The regional grouping reflects geographic similarities, whereas the temporal grouping allows for trend analysis across different periods.

In high-dimensional data analysis, data visualization plays a crucial preliminary role, with a rich history of development. To facilitate effective visualization, dimensionality reduction techniques are commonly employed. Linear methods such as principal components analysis (PCA; Hotelling [4]) and multidimensional scaling (MDS; Torgerson [5]) have been widely used. Moreover, several non-linear approaches have been developed to preserve the distances in the original high-dimensional space. These include stochastic neighbor embedding (SNE; Hinton and Roweis [6]), t-distributed SNE (t-SNE; Van Der Maaten and Hinton [7]), and uniform manifold approximation and projection (UMAP; McInnes et al. [8]).

Among various dimensionality reduction techniques, t-SNE stands out for its ability to capture local patterns by utilizing conditional probabilities to project high-dimensional data onto a lower-dimensional space. Notably, this study highlights the specific advantages of t-SNE in capturing complex local relationship within data, as demonstrated through numerous applications discussed in previous research by Amir et al. [9], Kobak and Berens [10], and Wang et al. [11]. On the computational side, Van Der Maaten [12] improved t-SNE's efficiency by using tree-based algorithms to approximate gradients, while Chan et al. [13] developed an optimized GPU implementation called t-SNE-CUDA for handling large-scale data visualization tasks.

In addition to its original formulation, several variations and extensions of t-SNE have been developed in the literature. For instance, Van Der Maaten [14] proposed a parametric version of t-SNE to learn parametric mapping. Gisbrecht et al. [15] and Gisbrecht et al. [16] proposed to represent the new observations by a linear combination of the low-dimensional representations and to use outcome information through a Fisher metric for a supervised version, respectively. More recently, Cheng et al. [17] introduced St-SNE, a novel supervised dimension reduction method preserving similarities in both feature and outcome spaces. Similarly, Meng et al. [18] introduced class-constrained t-SNE, which preserves similarities in both feature and class probability spaces. These approaches adopt a convex combination of multiple cost functions to accommodate multiple similarity measures. In these methods, each cost function is derived from t-SNE applied to a single similarity matrix and their combination enables the preservation of diverse structural relationships.

In this paper, we focus on developing a novel method namely Matrix t-SNE, specifically for visualizing high-dimensional matrix-framed data. Our goal is to find low-dimensional representations of elements in matrix-framed data that preserve both row-wise and column-wise group structures. For illustration, let us consider the task of reducing the dimensionality of an I×K matrix for visualization purpose. This can be achieved by flattening the matrix into a p=I⋅K-dimensional vector, where each element is labeled by a row index i=1,2,…,I and column index k=1,2,…,K. To achieve our goal of preserving both group structures, we define the distance between two elements $\left(i_{1},k_{1}\right)$ and $\left(i_{2},k_{2}\right)$ using both the row-wise distance $d_{r}(i_{1},i_{2})$ and the column-wise distance $d_{c}(k_{1},k_{2})$. Our approach involves finding a lower-dimensional representation of these *p* elements (i,k). There are two possible approaches to achieve this goal: (i) applying the classical t-SNE with a convex combination of row-wise and column-wise distances and (ii) optimizing a convex combination of two independent cost functions for the row-wise and column-wise structures, as inspired by Cheng et al. [17] and Meng et al. [18]. In this paper, we adopt the second approach (ii) to better preserve both row-wise and column-wise neighborhood structures by incorporating separate perplexities that account for the scale differences and variability of rows and columns. While the first approach is simpler than the second one by adopting a single perplexity, it is sensitive to scale differences between rows and columns. In contrast, the second approach accommodates these differences naturally, enabling a more balanced preservation of local neighborhoods in both row-wise and column-wise structures. For comparison, we also present the results of the first approach (i) in Appendix C.

Moreover, we can also view Matrix t-SNE as a projection into a low-dimensional space for enhanced biclustering of data elements. Biclustering is a statistical procedure that clusters the rows and columns of a matrix simultaneously, yielding groups where subsets of rows exhibit similar behavior across subsets of columns (or vice versa). For example, in bioinformatics, we can use biclustering to examine the dependencies between conditions (samples) and genes and identify condition-indicative genes. Traditional clustering methods might not be suitable for such cases, as genes may only exhibit similarity within specific conditions or vice versa. Biclustering encompasses various structural patterns, including exclusive row and column biclusters, and checkerboard structure. In exclusive row and column biclusters, every row and every column are assigned exclusively to one bicluster (Lazzeroni and Owen [19]). Checkerboard structures allow rows and columns to belong to more than one bicluster (Klug et al. [20]). Variations of these structures have been explored by researchers such as Tibshirani et al. [21] and Wang et al. [22]. However, Matrix t-SNE differs fundamentally from standard biclustering in its primary goal. While traditional biclustering aims to group elements into distinct biclusters, the main objective of Matrix t-SNE is dimension reduction to obtain a low-dimensional representation of high-dimensional data. This distinction enables Matrix t-SNE to provide a more comprehensive understanding of complex relationships within the data by preserving the structure and patterns in the original data.

The remainder of the paper is organized as follows. In Section 2, we introduce a novel t-SNE variant specifically designed for matrix-framed data and provide an algorithm to compute Matrix t-SNE. In Section 3, we demonstrate the effectiveness of our new method by applying it to three real data examples: visualization of skeleton joints and motion types using exergame data, visualization of mutation types and genes using breast cancer data, and visualization of regions and years using temperature data. Additionally, we provide a comparative analysis between Matrix t-SNE with the classical t-SNE with unstructured distance, highlighting the distinctions and advantages of our approach. Finally, in Section 4, we conclude the paper by summarizing its main findings and discussing potential avenues for further extension and exploration.

## 2 Method

### 2.1 Data and notation

Let us consider a matrix-framed dataset **X** consisting of *I* rows and *K* columns, where each element $X_{ik}$ represents the (i,k)-th element of **X**, $X_{i\cdot }$ represents the *i*-th row, and $X_{\cdot k}$ represents the *k*-th column for i=1,2,…,I, k=1,2,…,K. Here, we emphasize that the elements $X_{ik}$ are not restricted to scalars; they can represent arbitrary forms such as scalars, vectors, or matrices. For example, in the gene expression dataset discussed in Section 3, each element $X_{ik}\in R^{1}$ represents a scalar value, whereas in the exergame dataset, each $X_{ik}\in R^{T\times 3}$ corresponds to a three-dimensional (3D) trajectory of a joint.

We introduce Matrix t-SNE, a novel t-distributed stochastic neighborhood embedding method specifically designed for matrix-framed data. By embedding the matrix-framed data into a new space, Matrix t-SNE aims to better capture and represent the structure inherent in the matrix. Typically, we embed the data into a lower-dimensional, and without loss of generality, assume a two-dimensional space. The resulting embedded data is denoted by $Y_{ik}$, where $Y_{ik}=(Y_{ik1},Y_{ik2})\in R^{2}$. While embedding into a two-dimensional space may not constitute strict dimensionality reduction in cases like the gene expression dataset, it provides a more suitable embedding that better captures the matrix structure.

### 2.2 Matrix t-distributed stochastic neighborhood embedding

Matrix t-SNE minimizes the Kullback-Leibler divergence (7) between the joint probabilities in the high-dimensional space, $p_{ij}^{r}$ and $p_{kl}^{c}$, and their respective counterparts in the low-dimensional space, $q_{ij}^{r}$ and $q_{kl}^{c}$, for i,j=1,2,…,I and k,l=1,2,…,K. The superscripts ‘r’ and ‘c’ indicate the row-wise and column-wise embeddings, respectively. The pairwise similarity between subjects (rows) in the high-dimensional space $p_{ij}^{r}$ is defined as

(1) $$p_{ij}^{r}≔\frac{p_{j|i}^{r}+p_{i|j}^{r}}{2I}$$

where

(2) $$p_{j|i}^{r}≔\frac{\exp(-d{\left(X_{i\cdot },X_{j\cdot }\right)}^{2}/2\sigma_{i}^{2})}{\sum_{i^{\prime }\neq j^{\prime }}\exp(-d{\left(X_{i^{\prime }\cdot },X_{j^{\prime }\cdot }\right)}^{2}/2\sigma_{i}^{2})},$$

$d(X_{i\cdot },X_{j\cdot })$ denotes the distance between rows *i* and *j* in the high-dimensional space, and the variance $\sigma_{i}^{2}$ is chosen such that the conditional distribution $P_{i}^{r}$ achieves the desired perplexity $perp^{r}$, as explained in Van Der Maaten and Hinton [7]. Note that the calculation of distance is selected depending on the structures of elements. For instance, Van Der Maaten and Hinton [7] considered the Euclidean distance for scalar elements. In Section 3, for 3D trajectory elements in the exergame data, we consider the multi-dimensional dynamic time warping (DTW) distance (Shokoohi-Yetka et al. [23]) (i.e., $d{\left(X_{i\cdot },X_{j\cdot }\right)}^{2}=\sum_{k=1}^{K}\mathrm{DTW}{\left(X_{ik},X_{jk}\right)}^{2}$). For the breast cancer data, we use the Euclidean distance for scalar elements (i.e., $d{\left(X_{i\cdot },X_{j\cdot }\right)}^{2}=||X_{i\cdot }-X_{j\cdot }|{|}^{2}$). Lastly, for the temperature data, we use the Wasserstein (WS) distance (Irpino and Verde [24] and Kang et al. [25]) for histogram elements (i.e., $d{\left(X_{i\cdot },X_{j\cdot }\right)}^{2}=\sum_{k=1}^{K}\mathrm{WS}{\left(X_{ik},X_{jk}\right)}^{2}$). The row-wise perplexity captures the local neighborhood structure of rows in the high-dimensional space. Here, we use $P_{i}^{r}$ to represent the conditional probability across all data points, given the data point $X_{i\cdot }$. We define the corresponding pairwise similarities in the low-dimensional space $q_{ij}^{r}$ as

(3) $$q_{ij}^{r}≔\frac{{\left(1+\sum_{k=1}^{K}d{\left(Y_{ik},Y_{jk}\right)}^{2}\right)}^{-1}}{\sum_{i^{\prime }\neq j^{\prime }}{\left(1+\sum_{k=1}^{K}d{\left(Y_{i^{\prime }k},Y_{j^{\prime }k}\right)}^{2}\right)}^{-1}},$$

where $d(Y_{ik},Y_{jk})=||Y_{ik}-Y_{jk}||$ denotes the Euclidean distance between the two embeddings $Y_{ik}$ and $Y_{jk}$ in $R^{2}$, as defined in Section 2.1.

Similarly, we define the pairwise similarities between variables (columns) in the high-dimensional space $p_{kl}^{c}$ as

(4) $$p_{kl}^{c}≔\frac{p_{l|k}^{c}+p_{k|l}^{c}}{2K}$$

where

(5) $$p_{l|k}^{c}≔\frac{\exp(-d{\left(X_{\cdot k},X_{\cdot l}\right)}^{2}/2\sigma_{k}^{2})}{\sum_{k^{\prime }\neq l^{\prime }}\exp(-d{\left(X_{\cdot k^{\prime }},X_{\cdot l^{\prime }}\right)}^{2}/2\sigma_{k}^{2})},$$

and the variance $\sigma_{k}^{2}$ is determined by finding the value that yields a conditional probability $P_{k}^{c}$ for a given perplexity $perp^{c}$. This column-wise perplexity reflects the local neighborhood structure of columns in the high-dimensional space. The corresponding pairwise similarity in the low-dimensional space $q_{kl}^{c}$ is defined as

(6) $$q_{kl}^{c}≔\frac{{\left(1+\sum_{i=1}^{I}d{\left(Y_{ik},Y_{il}\right)}^{2}\right)}^{-1}}{\sum_{k^{\prime }\neq l^{\prime }}{\left(1+\sum_{i=1}^{I}d{\left(Y_{ik^{\prime }},Y_{il^{\prime }}\right)}^{2}\right)}^{-1}}.$$

Then we minimize a convex combination of the row-wise and column-wise cost functions, which are defined as the sum of corresponding KL divergences between $P^{r}$ and $Q^{r}$, and between $P^{c}$ and $Q^{c}$, respectively. We define each column (or row)-wise KL divergence by the distances between column (or row) vectors, and each distance is calculated as the sum of element-wise distances across rows (or columns). The combined cost function is

(7) $$C≔KL(P^{r},P^{c}|Q^{r},Q^{c})=\alpha \sum_{i=1}^{I}\sum_{j=1}^{I}p_{ij}^{r}\log\frac{p_{ij}^{r}}{q_{ij}^{r}}+(1-\alpha )\sum_{k=1}^{K}\sum_{l=1}^{K}p_{kl}^{c}\log\frac{p_{kl}^{c}}{q_{kl}^{c}}$$

where we set $p_{ii}^{r}=p_{ii}^{c}=q_{ii}^{r}=q_{ii}^{c}=0$ and the weight α∈[0,1]. However, the column (or row)-wise KL divergence is not simply the sum of element-wise KL divergences. Therefore, this combined cost function of Matrix t-SNE cannot be expressed as the sum of separable element-wise KL divergences, ensuring that both row-wise and column-wise relationships are preserved in the low-dimensional embedding and effectively capturing the joint structure of matrix-framed data.

In short, Matrix t-SNE extends the classical t-SNE to matrix-framed data. As in the classical t-SNE, our method derives joint probability distributions in the high-dimensional space, computes their corresponding probabilities in the low-dimensional representation, and minimizes the KL divergence between these two distributions. However, a crucial distinction exists between Matrix t-SNE and the classical t-SNE. In particular, the KL divergence formulation is modified to incorporate a convex combination of row-wise and column-wise KL divergences, each following the classical t-SNE formulation but applied separately to row-wise and column-wise distances. This innovative design enables both structural relationships inherent in the matrix-framed data to be preserved in the embedding space, thereby providing a more comprehensive representation of the underlying patterns.

The cost function (7) is minimized by a gradient descent approach, in which the gradient of the cost function is given by

(8) $$\frac{\partial C}{\partial Y_{ik}}=4\alpha^{2}\sum_{j=1}^{I}(p_{ij}^{r}-q_{ij}^{r}){\left(1+\sum_{k^{\prime }=1}^{K}d{\left(Y_{ik^{\prime }},Y_{jk^{\prime }}\right)}^{2}\right)}^{-1}(Y_{ik}-Y_{jk})+4{\left(1-\alpha \right)}^{2}\sum_{l=1}^{K}(p_{kl}^{c}-q_{kl}^{c}){\left(1+\sum_{i^{\prime }=1}^{I}d{\left(Y_{i^{\prime }k},Y_{i^{\prime }l}\right)}^{2}\right)}^{-1}(Y_{ik}-Y_{il}).$$

We provide a detailed derivation of the gradient in Appendix A.

As in Van Der Maaten and Hinton [7], we update the gradient with a momentum term and summarize the Matrix t-SNE algorithm in Algorithm 1. We use the same optimization parameters as in Van Der Maaten and Hinton [7]: iteration M=1000; momentum γ(m)=0.5 for m<250 and γ(m)=0.8 for m≥250; initial learning rate η=100. Since α=0 and α=1 correspond to the original t-SNE, we use equal initial scores $Y_{ik}^{\left(0\right)}$ across columns k=1,2,…,K for fixed *i* when α=1 and across rows i=1,2,…,I for fixed *k* when α=0, respectively. For α∈(0,1), we initialize $Y_{ik}$ using a standard Gaussian distribution. To account for the distinct neighborhood structures of rows and columns, Matrix t-SNE incorporates two independently defined perplexity parameters, $perp^{r}$ and $perp^{c}$. These parameters adapt to the local distance distributions of rows and columns, respectively. By normalizing pairwise similarities into probabilities, these perplexities achieve scale invariance while preserving row-wise and column-wise group structures.

**Algorithm 1:**
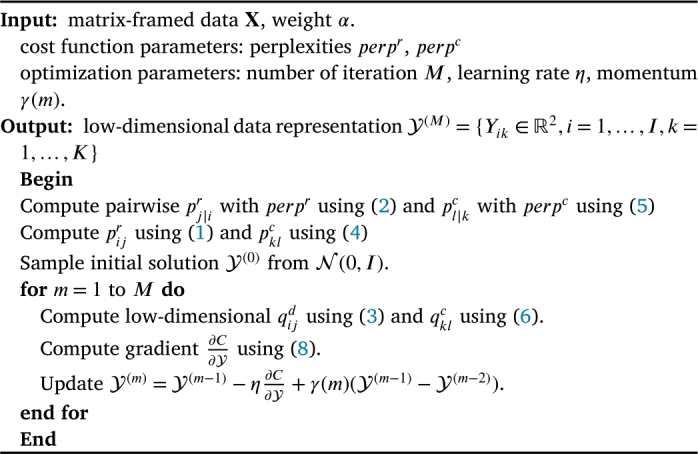
Matrix t-SNE algorithm.

### 2.3 Selection of weight *α*

The minimization of the KL divergence (7) yields I⋅K data points, which are achieved through the simultaneous embedding of rows and columns. The weight *α* leverages the importance of row-wise and column-wise variations in separating the data points. In particular, the special cases α=0 and α=1 correspond to applying t-SNE to the column vectors and row vectors, respectively. In practice, the choice of the weight *α* should balance both column and row structures of the matrix data. Here we adopt the following procedure for selecting *α*.

Let $G_{0}$ and $G_{1}$ represent the number of clusters in the embeddings obtained from column-wise (α=0) and row-wise (α=1) t-SNE, respectively. In our implementation, we define these quantities as

$$G_{0}=\min\left\{g:\frac{SSB_{0}(g)}{SST_{0}(g)}>0.9\right\}\;\;\text{and}\;G_{1}=\min\left\{g:\frac{SSB_{1}(g)}{SST_{1}(g)}>0.9\right\},$$

where $SSB_{\alpha }(g)$ and $SST_{\alpha }(g)$ represent the sum of squares between clusters and the total sum of squares for *g* clusters under a given *α*, respectively. Although we employed K-means clustering to determine $G_{0}$ and $G_{1}$ in this article, alternative clustering methods could also be used for this purpose. We then define the true clusters as the combination of marginal clusters from separate column-wise and row-wise embeddings. Consequently, the total number of clusters is set as $G=G_{0}\times G_{1}$ for Matrix t-SNE. Given these *G* clusters, we define the optimal weight $\alpha^{⁎}$ as

(9) $$\alpha^{⁎}=\arg\max_{\alpha \in (0,1)}\frac{SSB_{\alpha }(G)}{SST_{\alpha }(G)}.$$

This approach naturally aligns with the goal of Matrix t-SNE to simultaneously preserve both marginal structures, ensuring that the overall variability of the matrix-framed data is best explained. While our primary selection metric is the SSB/SST ratio, alternative clustering metrics such as Adjusted Rand Index (ARI) and Normalized Mutual Information (NMI) may also be employed for selecting $\alpha^{⁎}$.

## 3 Examples and applications

To illustrate Matrix t-SNE, we apply it to three real datasets, the exergame data, the breast cancer data, and temperature data. We compare the results with the classical t-SNE, which creates low-dimensional embeddings of elements using unstructured distance (without consideration of row-wise or column-wise structure). For this comparison, we computed unstructured pairwise distances directly from the high-dimensional data using a suitable distance metric $d(X_{ik},X_{jl})$. For instance, we used DTW distance $\mathrm{DTW}(X_{ik},X_{jl})$ for the exergame data, Euclidean distance $\left||X_{ik}-X_{jl}|\right|$ for the breast cancer data, and Wasserstein distance $\mathrm{WS}(X_{ik},X_{il})$ in temperature data.

The computational complexity of Matrix t-SNE is O(IK(I+K)), whereas for the classical t-SNE with unstructured distance, it is $O({\left(IK\right)}^{2})$. As matrix data size increases, both Matrix t-SNE and t-SNE become slower, but t-SNE becomes significantly slower compared to Matrix t-SNE. For instance, when I=K, Matrix t-SNE has cubic complexity $O(I^{3})$, whereas t-SNE scales with the fourth power $O(I^{4})$, making Matrix t-SNE significantly more efficient for large matrix data. For the exergame dataset (40×25), Matrix t-SNE completed in 1.09 seconds, while t-SNE took 11.03 seconds. For the breast cancer dataset (15×176), Matrix t-SNE completed in 6.08 seconds, compared to 81.01 seconds for t-SNE. For the temperature dataset (5×55), Matrix t-SNE completed in 0.41 seconds, while t-SNE took 0.88 seconds.

**Fig. 1:**
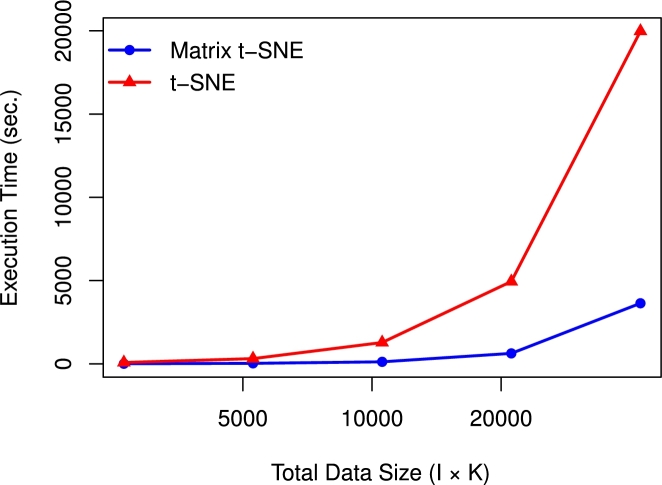
Execution time (in seconds) for different data sizes using the breast cancer dataset for the fixed *I* = 15 and varying *K*.

To further investigate the computational complexity and scalability for larger-scale datasets, we conducted a sensitivity analysis using the breast cancer dataset. Specifically, we increased the number of genes (*K*) while keeping the number of subjects (*I*) fixed by bootstrapping the genes (column vectors), and recorded the execution time in seconds for both Matrix t-SNE and the classical t-SNE. We increased *K* exponentially, resulting in matrix sizes of I×K,I×2K,I×4K,I×8K, and I×16K. Consequently, the total number of embedded data points reached 2640,5280,10560,21120, and 42240. The results are presented in Fig. 1 and show that Matrix t-SNE can be done in substantially lower computation time compared to t-SNE as the dataset size increases. This finding demonstrates that Matrix t-SNE can handle larger structured datasets efficiently, making it computationally feasible for large-scale applications. All analyses were performed on a PC with an Intel(R) Xeon(R) W-2145 CPU @ 3.70GHz and 64GB of memory.

**Fig. 2:**
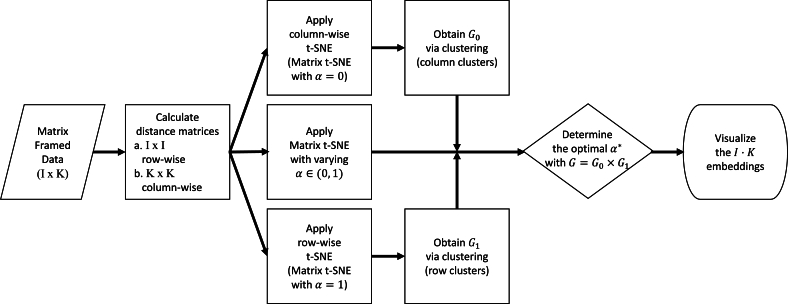
Flowchart of the Matrix t-SNE.

**Fig. 3:**
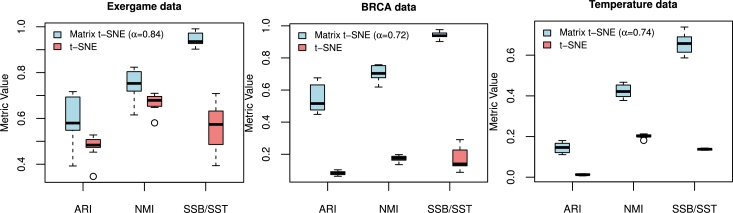
Boxplots of quantitative performance comparisons across three metrics (ARI, NMI, SSB/SST) for Matrix t-SNE and t-SNE at the selected *α*, chosen based on SSB/SST for each dataset.

Through these examples, we aim to visualize the data points based on different attributes including motion types and joint skeletons for the exergame data, mutation types and genes for the breast cancer data, and regions and years for temperature data. With the optimal weight $\alpha^{⁎}$ derived based on Section 2.3, we find the representation that captures both the column-wise and row-wise structures underlying the data. The process of applying Matrix t-SNE is depicted in Fig. 2. Additionally, we demonstrate how Matrix t-SNE provides a more comprehensive representation of matrix-framed data compared to the classical t-SNE with unstructured distance. To quantitatively assess the robustness and stability of Matrix t-SNE, we compared its performance with t-SNE by computing three performance metrics-ARI, NMI, and SSB/SST-to evaluate clustering quality and embedding separability using the combined clusters defined from the marginal embeddings, as described in Section 2.3. The experiments were conducted using the selected weight $\alpha^{⁎}$, which was set to 0.84, 0.72, and 0.74 for the exergame, breast cancer, and temperature datasets, respectively. We evaluated each method over 10 perturbed maps generated from different random initializations. The results, summarized as boxplots in Fig. 3, indicate that Matrix t-SNE consistently outperforms t-SNE across all datasets and evaluation metrics. This demonstrates its effectiveness in preserving meaningful structures in the embedded space.

When the group memberships are already known for each feature (row or column), we skip the K-means clustering for α=0 or 1 and directly use the known groups to define $G_{0}$ and $G_{1}$, as outlined in Section 2.3. For instance, in our exergame data example, we utilize both joint and motion groups which are already known. In contrast, for the breast cancer data, we use the known mutation information (BRCA1 and BRCA2) but employ K-means clustering to identify gene groups through from the genes' embeddings. Notably, on these examples, even though the group memberships for rows or columns are already known, we applied the process outlined in the flowchart to identify these memberships, and the resulting groupings are closely match the actual memberships.

**Fig. B.8:**
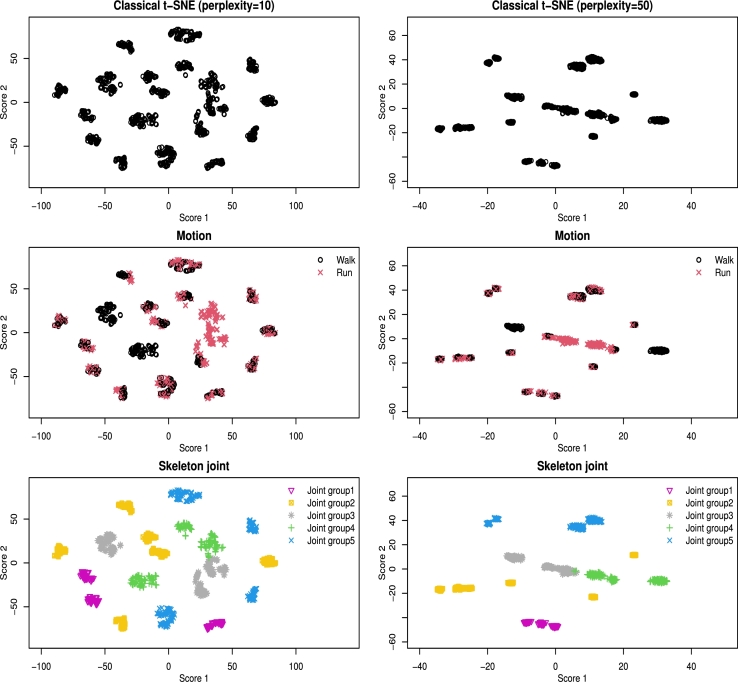
Impact of perplexity on embeddings using the classical t-SNE with unstructured distance on the exergame dataset (40 × 25). Top panels: embeddings without color differentiation; middle panels: colored by motion type; bottom panels: colored by skeleton joint group.

**Fig. B.10:**
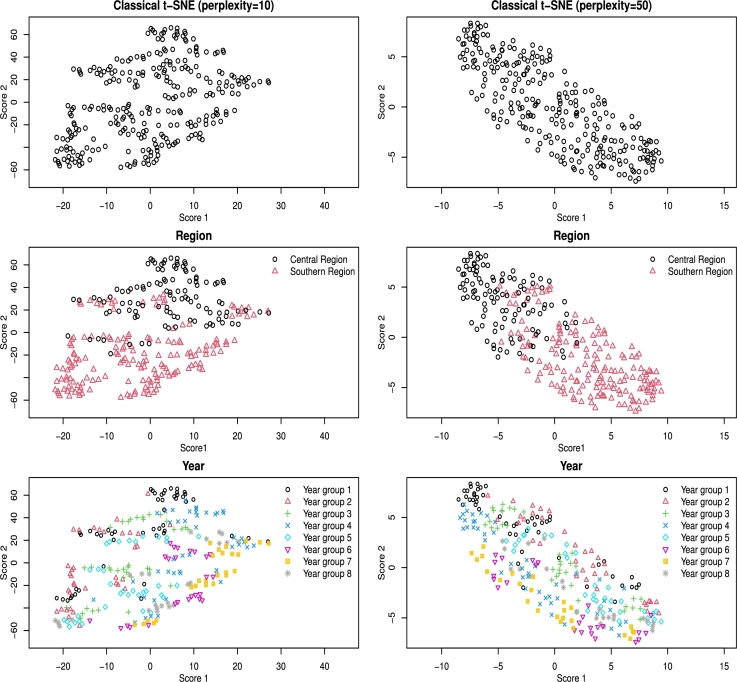
Impact of perplexity on embeddings using the classical t-SNE with unstructured distance on the temperature dataset (5 × 55). Top panels: embeddings without color differentiation; middle panels: colored by region group; bottom panels: colored by year group.

For Matrix t-SNE, we set two perplexity values based on recommendations from the R package Rtsne. Specifically, the perplexity for row-wise t-SNE (α=1) and column-wise t-SNE (α=0) were chosen as the smaller of one-third of the sample size or the default value of 30. To preserve the marginal structures in both row and column spaces, Matrix t-SNE directly adopts these values for corresponding perplexities, $perp^{r}$ and $perp^{c}$, respectively. For the classical t-SNE with unstructured distance, we similarly used the default perplexity value of 30, and the results using various perplexity values are provided in Fig. B.8, Fig. B.10 in Appendix B.

### 3.1 Exergame data

**Fig. 4:**
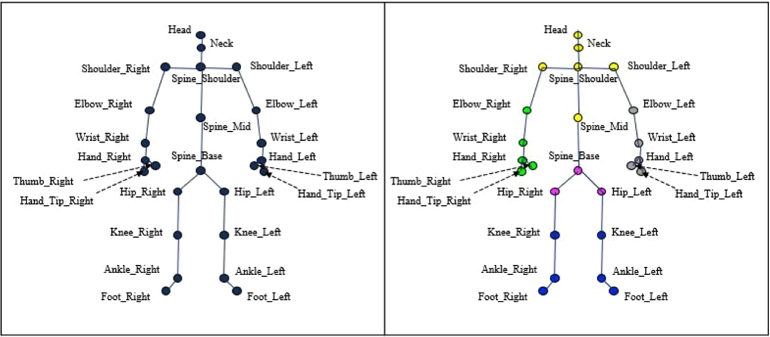
25 Skeletons by Microsoft Kinect v2 (left) and 5 clusters of skeletons (right).

We apply Matrix t-SNE to the exergame data collected from a physical activity study conducted by Kim et al. [26] investigating the effects of exercise type and gameplay mode. This dataset consists of skeleton joint sequence data captured by the Microsoft Kinect V2 sensor. Microsoft Kinect is a sophisticated sensor that localizes human body parts and produces moving 3D skeletons for action recognition. The dataset comprises 3D coordinate (x,y,z) for 25 joints, continuously provided at 30 frames per second. Fig. 4 shows the 25 joints. The camera's center point is set as the origin (x=0,y=0,z=0), with the x-axis representing left and right movements, the *y*-axis up and down movements, and the *z*-axis front and back depths.

A total of 20 participants performed walking and running motions for 3 minutes each motion while playing a Nintendo Switch game, resulting in 5400 frames (=3×60 sec. ×30 frames/sec.) of observed skeleton coordinates per motion. For each motion, 60 frame-length joint information was extracted from the data observed for 2 seconds. In detail, the point where the left foot is closest to the ground was set as the starting point so that there were no overlapping frames for each motion. Then, 60 frames from each starting point generated a sequence, resulting in approximately 70 sequences for each subject's motion. After preprocessing (i.e., averaging the sequences on each subject's motion), we obtained 60 frame-length 3D coordinates for 25 joints for each participant's motion. Readers can refer to Kim et al. [26] for detailed information on the data and preprocessing steps.

To apply Matrix t-SNE to this exergame data, we extend the notations as follows: we denote the (i,k)-th element $X_{ik}=(X_{ik}^{x},X_{ik}^{y},X_{ik}^{z})$ where $X_{ik}^{p}=\{X_{ik}^{p}(t),t=1,2,\ldots ,T\}$ for p=x,y,z and $\left(X_{ik}^{x}(t),X_{ik}^{y}(t),X_{ik}^{z}(t)\right)$ is the *k*-th skeleton joint's 3D coordinate of *i*-th subject at the *t*-th frame (time) for i=1,2,…,I=40(=20×2), k=1,2,…,K=25, and t=1,2,…T=60. Thus, each (i,k)-th element is a three-dimensional vector measured across times (i.e., multivariate longitudinal data). In other words, the size of the matrix data is 40×25 where each entry is a three-dimensional vector across 60 time points. Accordingly, we consider the multi-dimensional DTW distance (Shokoohi-Yetka et al. [23]) in the probabilities $p_{j|i}^{d}$ and $p_{j|i}^{c}$. To be specific, in (2), the distance between skeleton joint sequences of the *i*-th and *j*-th subjects is given by

(10) $$d{\left(X_{i\cdot },X_{j\cdot }\right)}^{2}=\sum_{k=1}^{K}\mathrm{DTW}{\left(X_{ik},X_{jk}\right)}^{2}=\sum_{k=1}^{K}\{\mathrm{DTW}{\left(X_{ik}^{x},X_{jk}^{x}\right)}^{2}+\mathrm{DTW}{\left(X_{ik}^{y},X_{jk}^{y}\right)}^{2}+\mathrm{DTW}{\left(X_{ik}^{z},X_{jk}^{z}\right)}^{2}\}.$$

Similarly, in (5), the distance between motions of the *k*-th and *l*-th skeleton joints is given by

(11) $$d{\left(X_{\cdot k},X_{\cdot l}\right)}^{2}=\sum_{i=1}^{I}\mathrm{DTW}{\left(X_{ik},X_{il}\right)}^{2}=\sum_{i=1}^{I}\{\mathrm{DTW}{\left(X_{ik}^{x},X_{il}^{x}\right)}^{2}+\mathrm{DTW}{\left(X_{ik}^{y},X_{il}^{y}\right)}^{2}+\mathrm{DTW}{\left(X_{ik}^{z},X_{il}^{z}\right)}^{2}\}.$$

The pairwise DTW distances between sequences were computed using the dtw package in R with the default alignment setting (window.type=”none”), which allows full flexibility in sequence alignment.

**Fig. 5:**
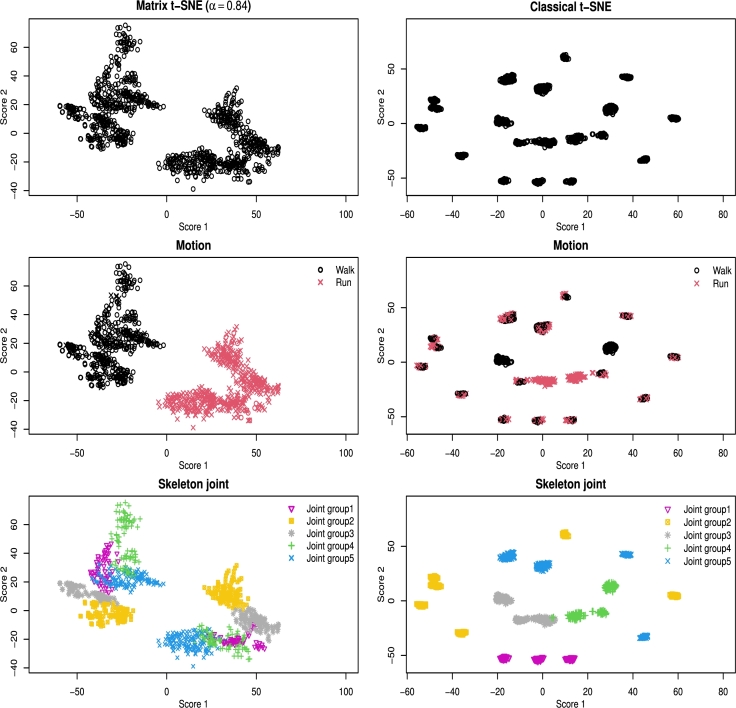
Visualization of exergame data (40 × 25) by Matrix t-SNE at a selected weight (*α*^⁎^ = 0.84) and the classical t-SNE with unstructured distance. Top panels: embeddings without color differentiation; middle panels: colored by motion type; bottom panels: colored by skeleton joint group.

To find a low-dimensional representation of the data points, we first identify the column-wise structure (the clusters of skeleton joints) and the row-wise structure (the clusters of motions) by applying the original t-SNE which corresponds to Matrix t-SNE with α=0 and α=1, respectively. As a result, we identified $G_{0}=5$ and $G_{1}=2$, which matched the actual memberships, and set G=10. For this selected *G*, we determined the optimal weight $\alpha^{⁎}$ using (9) as $\alpha^{⁎}=0.84$. The subjects were clustered based on their motions, and the skeleton joints were grouped into five subgroups according to their anatomical locations. These group memberships are indicated by different colors in Fig. 4, Fig. 5, which display the results obtained using Matrix t-SNE. In addition, an alternative analysis using alternative clustering metrics ARI and NMI resulted in a slightly different optimal weight of $\alpha^{⁎}=0.78$. The corresponding embedding visualizations are provided in Appendix D and show minimal differences from those obtained with $\alpha^{⁎}$, indicating the stability of the embeddings across different metric-based selections of *α*.

The left three panels in Fig. 5 illustrate the 40×25 embeddings generated by Matrix t-SNE at the optimal weight $\alpha^{⁎}=0.84$. Two additional figures are presented: one colored by motion type (middle) and another by skeleton joint group (bottom). The middle panel shows a clear separation of data points based on the two motions, while the bottom panel shows how the joint groups are clustered within each motion type. These figures effectively visualize both column-wise and row-wise structures. Specifically, the joints are well separated in the walking motion but partially overlap in the running motion. This indicates that the nested structures differ depending on the motion type.

In contrast, the classical t-SNE with unstructured pairwise DTW distance is applied directly to the 3D trajectory elements without considering row and column structures. The right three panels in Fig. 5 show the resulting embeddings, with data points colored by motion type (middle) and skeleton joint group (bottom). The bottom panel shows the given embedding separates finely the skeleton joint group than the marginal row-wise embedding. However, it visualizes well different skeleton joint groups. The middle panel shows the embedding colored by motion types, where two motion types are not differentiated clearly. This result is because the variability of the distances among different skeleton joints is smaller than that between motions, and thus, the local neighbors in this case more easily preserves the group structure of the skeleton joints than the motions.

### 3.2 Breast cancer data

Next, we consider the breast cancer dataset from a gene expression study of BRCA tumors in 22 beast cancer patients as reported in [27]. The breast cancers are associated with three types of mutations: BRCA1, BRCA2, and Sporadic mutations. Seven patients have the BRCA1 mutation, eight patients have the BRCA2 mutation, and the remaining seven patients have the sporadic type. For each patient, 3226 genes are observed, resulting in a 22×3226 matrix data. To illustrate the new method, we selected the most significant 176 genes that could discriminate breast cancers with BRCA1 mutation from those with BRCA2 mutation, resulting in a 15×176 matrix. The selected 176 genes are listed in the supplementary material of [27]. Note that we used the Euclidean distance in the probabilities $p_{j|i}^{d}$ and $p_{l|k}^{c}$. The data had been preprocessed through log transformation and normalization.

**Fig. 6:**
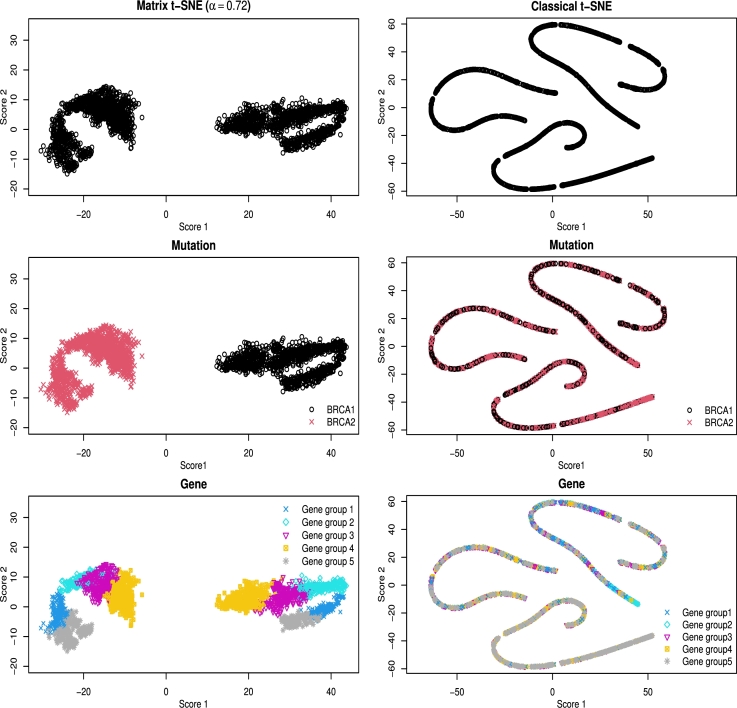
Visualization of breast cancer data (15 × 176) by Matrix t-SNE at a selected weight (*α*^⁎^ = 0.72) and the classical t-SNE with unstructured distance. Top panels: embeddings without color differentiation; middle panels: colored by mutation type; bottom panels: colored by gene group.

Using row-wise t-SNE, the data points are separated clearly $G_{1}=2$ groups, corresponding to the two actual mutation types. Using column-wise t-SNE, we identified that the genes clustered into $G_{0}=5$ groups. Therefore, we set G=10 and determined the optimal weight $\alpha^{⁎}=0.72$ using (9). The Matrix t-SNE embeddings are shown in Fig. 6. An alternative analysis with ARI and NMI yielded a similar optimal weight $\alpha^{⁎}=0.68$, confirming the stability of embeddings (see Appendix D).

The left three panels in Fig. 6 illustrate the 15×176 embeddings generated by Matrix t-SNE at the optimal weight $\alpha^{⁎}=0.72$. The middle panel is colored by mutation type and the bottom panel is colored by gene cluster. The middle panel shows a clear separation of data points based on the two mutation types, while the bottom panel illustrates how the gene groups are clustered within each mutation type. These figures effectively visualize both column-wise and row-wise structures. Specifically, the gene groups are well separated in both BRCA1 and BRCA2 mutations, although the BRCA2 genes are more densely clustered. This indicates that the nested structures are similar depending on the mutation type.

In contrast, the right three panels in Fig. 6 display the results from the classical t-SNE with unstructured distance. The resulting embeddings show a tangled structure, lacking clear separation of both mutation types (middle) and gene groups (bottom). Due to the scalar nature of the elements in this matrix, the distances between elements are simply Euclidean distances between points, leading to the formation of three elongated bands based solely on straight-line distances: near zero, medium, and long. These bands do not reflect any meaningful structural relationships. The gene clusters and mutation types are less apparent, highlighting the limitation of the classical t-SNE with unstructured distance in simultaneously preserving the underlying structures of genes and mutations, which Matrix t-SNE effectively captures.

### 3.3 Temperature data

To further assess the versatility of Matrix t-SNE, we examine a climate dataset that captures temperature variations across different regional and temporal units in Korea. The dataset contains daily average temperature records collected over a 55-year period, from January 1, 1969, to December 31, 2023, across five key regions: Seoul, Busan, Daegu, Gwangju, and Daejeon. We obtain the data from the Open MET Data Portal of the Korea Meteorological Administration (https://data.kma.go.kr/cmmn/main.do).

For each region and year, we summarize the daily temperature observations as a histogram, representing the distribution of temperatures over 365 days. Thus, the (i,k)-th element of the matrix is a histogram-valued entry. The dataset is structured as a 5×55 matrix, where each row corresponds to a region, each column represents a year, and each element is a histogram. To measure dissimilarities between elements, we use the Wasserstein distance (Irpino and Verde [24] and Kang et al. [25]), which is well-suited for comparing histogram-valued data, in the probabilities $p_{j|i}^{r}$ and $p_{l|k}^{c}$.

We employed row-wise t-SNE, which revealed two regional groups $G_{1}=2$: Group 1 (Central Region), which includes Seoul and Daejeon, and Group 2 (Southern Region), which consists of Busan, Daegu, and Gwangju. Conversely, using column-wise t-SNE, we identified $G_{2}=8$ distinct temporal clusters, which represent different climate patterns across historical periods. The specific year groupings are summarized in Table 1. Overall, the temporal clusters can be broadly categorized into two groups: Group 1-4 (pre-2000 dominant) and Group 5-8 (post-2000 dominant), reflecting the general increase in temperature around the year 2000. Notably, certain years before 2000 that exhibited higher temperatures - such as 1989, 1990, 1994, 1997, 1998, and 1999 - were grouped with post-2000 clusters (Group 5-8). Conversely, some post-2000 years that experienced relatively lower temperatures - including 2000, 2001, 2003, 2005, 2010, 2011, 2012, and 2013 - were classified with the earlier period clusters (Group 1-4). This pattern suggests that temperature variations played a key role in the clustering process, rather than a strict chronological separation. This temporal clustering effectively captures long-term climate trends and structural shifts in temperature distributions.

**Table 1:** Identified temporal clusters using column-wise t-SNE. Each temporal group represents a distinct climate pattern across historical periods.

Temporal Group	Years
Year group 1	1969, 1970, 1971, 1973, 1974, 1976, 1980, 1981, 1984, 1986
Year group 2	1972, 1979, 1987, 1989, 1991, 1992, 1993, 2003
Year group 3	1975, 1982, 1983, 1985, 1995, 1996, 2000
Year group 4	1977, 1978, 1985, 2001, 2005, 2010, 2011, 2012, 2013
Year group 5	1989, 1997, 1999, 2002, 2006, 2007, 2020
Year group 6	1990, 1998, 2004, 2021, 2023
Year group 7	1994, 2016, 2017, 2018, 2022
Year group 8	2008, 2009, 2014, 2015, 2019

**Fig. 7:**
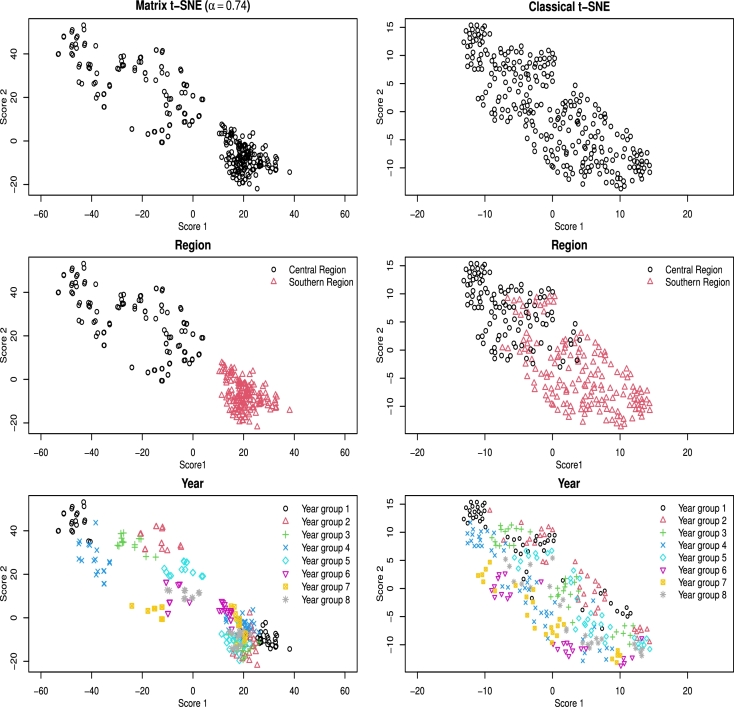
Visualization of temperature data (5 × 55) by Matrix t-SNE at a selected weight (*α*^⁎^ = 0.84) and the classical t-SNE with unstructured distance. Top panels: embeddings without color differentiation; middle panels: colored by regional group; bottom panels: colored by temporal group.

To determine the optimal weight $\alpha^{⁎}$, we set G=16 and obtained an optimal value of $\alpha^{⁎}=0.74$ using (9). The Matrix t-SNE embeddings are shown in Fig. 7. The left three panels in Fig. 7 illustrate the 5×55 embeddings generated by Matrix t-SNE at the optimal weight $\alpha^{⁎}=0.74$. The middle panel colors data points by regional groups, while the bottom panel highlights the clustering of years within each region. These visualizations effectively capture both row-wise (regional) and column-wise (temporal) structures. Notably, the temporal clusters are well-separated for Seoul and Daejeon (central region) but less distinct for Busan, Daegu, and Gwangju (southern region), suggesting that temperature variation patterns differ between these spatial groups. An alternative analysis with ARI and NMI yielded a similar optimal weight $\alpha^{⁎}=0.64$, confirming the stability of embeddings (see Appendix D).

In contrast, the right three panels in Fig. 7 display the results from the classical t-SNE using unstructured pairwise Wasserstein distance, with data points colored by regional group (middle) and temporal group (bottom). The middle panel shows that the regional groups are more finely separated than in the marginal column-wise embedding. However, the temporal clusters in the bottom panel are not differentiated. This suggests that while the classical t-SNE effectively captures regional variations, it struggles to differentiate temporal patterns due to the variability of distances among different regions being smaller than those among years. Consequently, local neighborhoods in this case tend to preserve regional structures rather than temporal structures.

## 4 Conclusion

In this paper, we propose a novel t-SNE procedure, named Matrix t-SNE, designed to visualize matrix data while preserving the inherent grouping patterns along both rows and columns. The workflow of Matrix t-SNE is depicted in Fig. 2. To demonstrate its effectiveness, we apply Matrix t-SNE to three real-world datasets, exergame data, breast cancer gene expression data, and temperature data. In addition, we compare it with the classical t-SNE with unstructured distance that disregards row and column structures in the data. Our results show that Matrix t-SNE maintains structural coherence in the embedded space while achieving comparable or improved computational efficiency relative to the classical t-SNE. Moreover, quantitative comparisons using clustering metrics (ARI, NMI, and SSB/SST) demonstrate that Matrix t-SNE consistently captures marginal structures more effectively across all datasets considered.

One notable feature of our Matrix t-SNE is its ability to preserve the nested dichotomy of the data. From the examples presented, we find that Matrix t-SNE maintains a hierarchical structure, where columns (or rows) form larger clusters and rows (or columns) form smaller clusters within these larger clusters. For instance, in the exergame data, the (smaller) clusters of joints are nested within the (larger) clusters of subjects (motions). Similarly, in the breast cancer data, gene clusters are nested within patient clusters (mutations). In contrast, the classical t-SNE with unstructured distance potentially tends to lose the inherent row-column structure (or either one of them) of the original matrix.

Matrix t-SNE effectively preserves both row-wise and column-wise structures regardless of scale differences. However, it has limitations. First, defining marginal structures such as $G_{0}$, $G_{1}$, and combined clusters is necessary to determine the optimal weight *α*. In this paper, we use K-means clustering for this purpose, constructing true clusters via the cross-product of marginal clusters. While K-means is computationally efficient and widely used, it assumes spherical clusters and may introduce bias when clusters are non-spherical or overlapping. Exploring alternative clustering methods, such as hierarchical clustering or density-based clustering, could capture more complex structures. This remains an open question for future research. Second, our current study does not include explicit experiments under conditions of noisy data and overlapping clusters. Similar to the classical t-SNE, Matrix t-SNE incorporates data-specific distance metrics (e.g., DTW, Wasserstein) as marginal distance metrics, which may provide greater robustness under conditions of noisy data and overlapping clusters. Therefore, we expect the selection of appropriate distance can mitigate the effects of noise and overlapping clusters. This also warrants further investigation.

Beyond the datasets analyzed in this study, Matrix t-SNE can be applied to various structured datasets where preserving both row-wise and column-wise dependencies is essential. For example, in electrogastrography (EGG) data, rows represent individuals or disease types, while columns correspond to frequency bands. Similarly, in longitudinal health data, such as blood pressure measurements over time, rows represent measurement types, and columns represent time points. These examples illustrate the versatility of Matrix t-SNE as a visualization tool for structured matrix-framed data across various domains.

## Code availability

The R code to fit Matrix t-SNE and example code for the breast cancer data are available at https://github.com/shahn63/Matrix-t-SNE. The repository includes a README file that provides detailed descriptions of all included files, their usage, example code, and step-by-step instructions on loading the necessary scripts and running Matrix t-SNE.

## CRediT authorship contribution statement

**Soohyun Ahn:** Writing – review & editing, Writing – original draft, Software, Methodology, Formal analysis. **Johan Lim:** Writing – review & editing, Writing – original draft, Methodology, Conceptualization. **Wei Jiang:** Writing – review & editing. **Sungim Lee:** Writing – review & editing, Visualization, Investigation. **Xinlei Wang:** Writing – review & editing, Supervision, Project administration.

## Declaration of Competing Interest

No conflict of interest is declared for this submission.

## Figures and Tables

**Fig. B.9:**
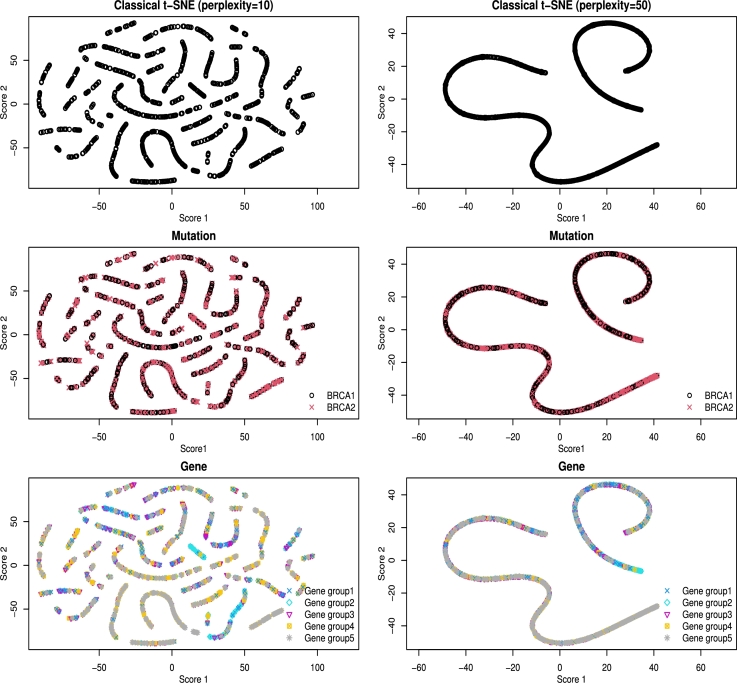
Impact of perplexity on embeddings using the classical t-SNE with unstructured distance on the breast cancer dataset (15 × 176). Top panels: embeddings without color differentiation; middle panels: colored by mutation type; bottom panels: colored by gene group.

**Fig. C.11:**
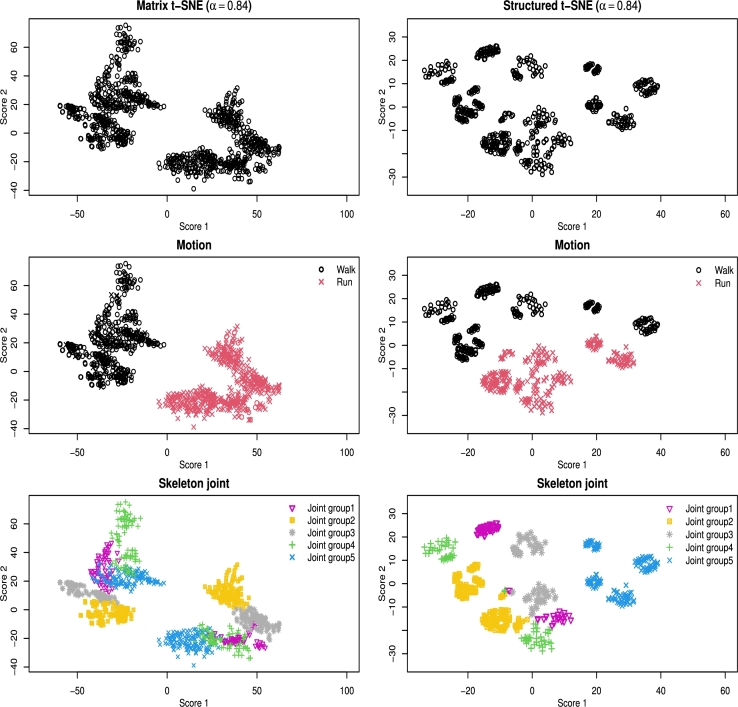
Comparison of (i) classical t-SNE with combined distance at a selected weight *α*^⁎^ = 0.84 and Matrix t-SNE at a selected weight *α*^⁎^ = 0.84 on the exergame dataset (40 × 25). Top panels: embeddings without color differentiation; middle panels: colored by motion type; bottom panels: colored by skeleton joint group.

**Fig. C.12:**
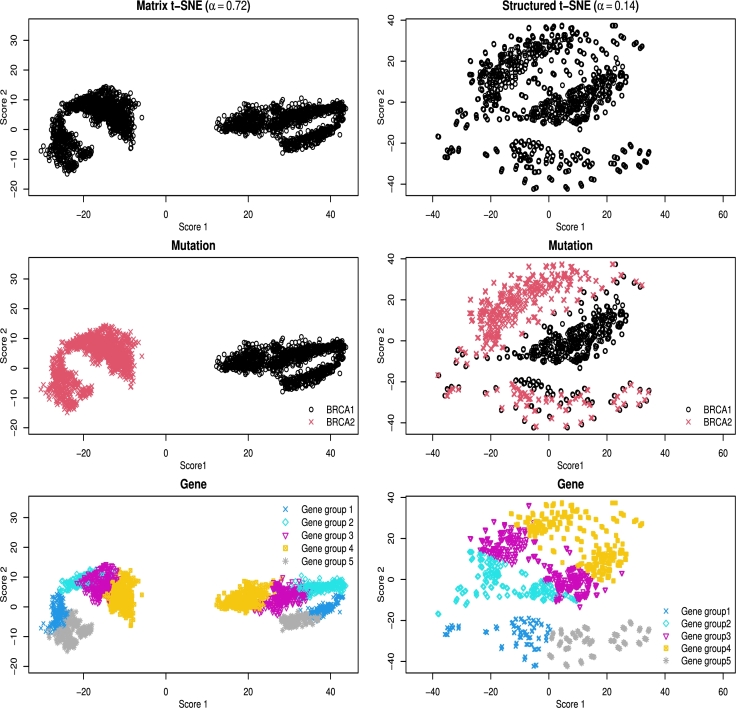
Comparison of (i) classical t-SNE with combined distance at a selected weight *α*^⁎^ = 0.14 and Matrix t-SNE at a selected weight *α*^⁎^ = 0.72 on the breast cancer dataset (15 × 176). Top panels: embeddings without color differentiation; middle panels: colored by mutation type; bottom panels: colored by gene group.

**Fig. C.13:**
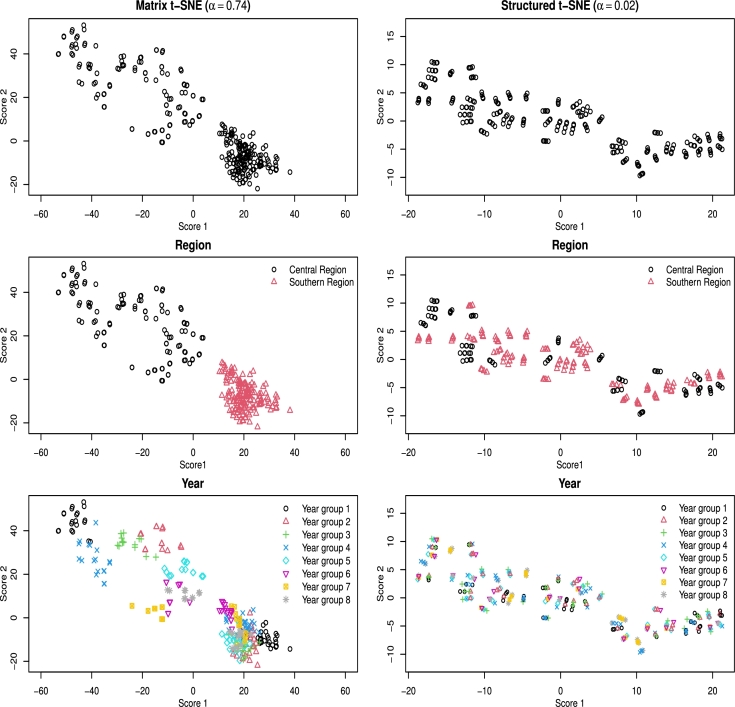
Comparison of (i) classical t-SNE with combined distance at a selected weight *α*^⁎^ = 0.02 and Matrix t-SNE at a selected weight *α*^⁎^ = 0.74 on the temperature dataset (5 × 55). Top panels: embeddings without color differentiation; middle panels: colored by region group; bottom panels: colored by year group.

**Fig. D.14:**
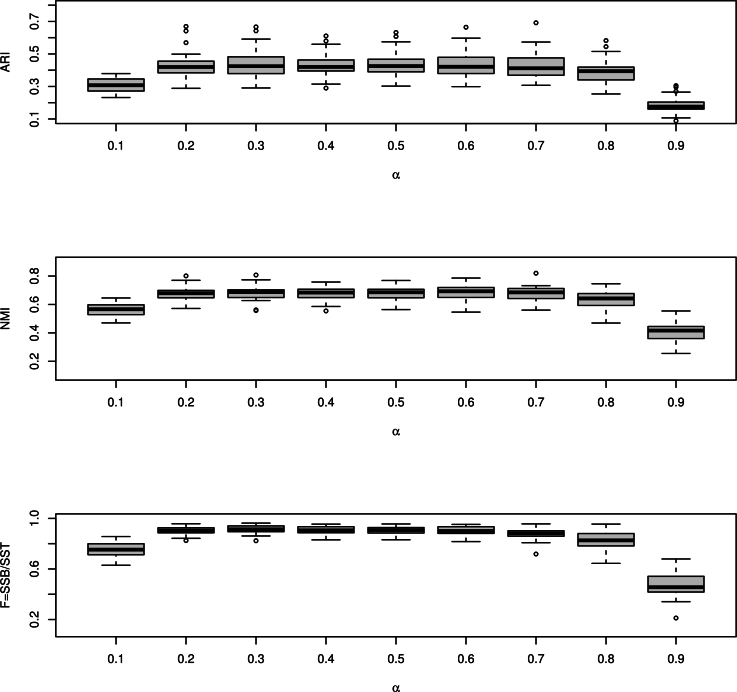
Boxplots of performance measures (ARI, NMI, and SSB/SST) across different *α* values over 30 bootstrapped datasets for the exergame dataset (40 × 25) using Matrix t-SNE. For the real dataset, the selected *α* values, *α*^⁎^ = 0.84 based on SSB/SST and *α*^⁎^ = 0.78 based on ARI and NMI.

**Fig. D.15:**
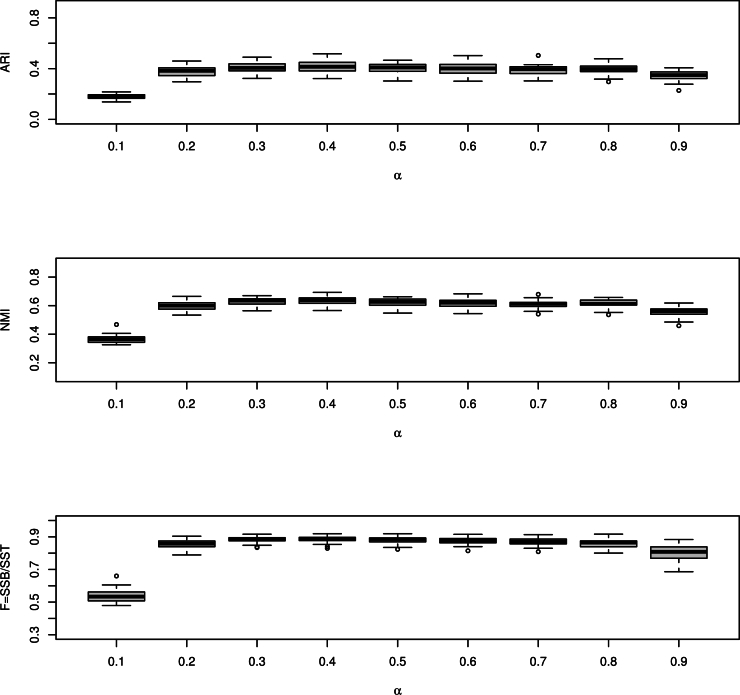
Boxplots of performance measures (ARI, NMI, and SSB/SST) across different *α* values over 30 bootstrapped datasets for the BRCA dataset (15 × 176) using Matrix t-SNE. For the real dataset, the selected *α* values are *α*^⁎^ = 0.72 based on SSB/SST and *α*^⁎^ = 0.68 based on ARI and NMI.

**Fig. D.16:**
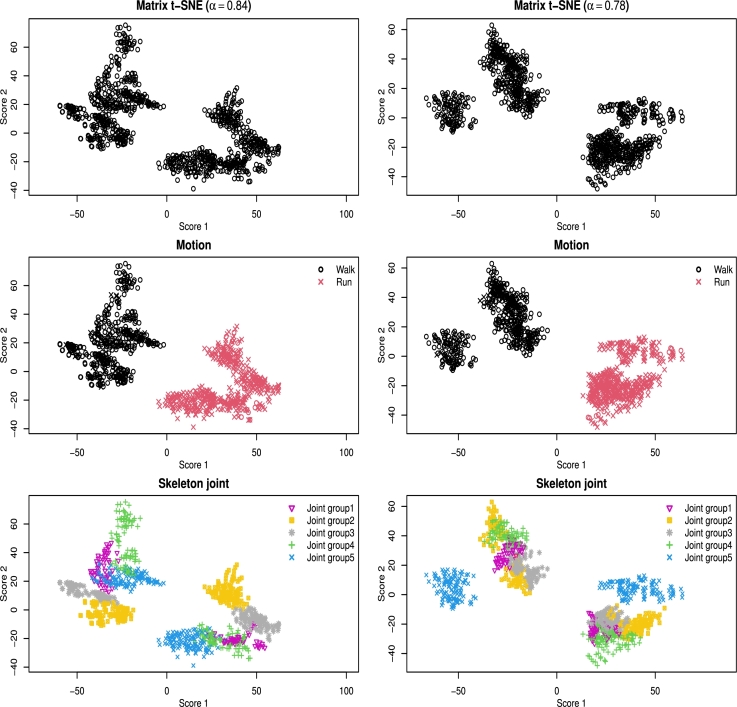
Impact of *α* selection on embeddings for the exergame dataset (40 × 25) using Matrix t-SNE at *α*^⁎^ = 0.84 chosen based on the *SSB*/*SST* metric and *α*^⁎^ = 0.78 chosen based on ARI and NMI metrics.

**Fig. D.17:**
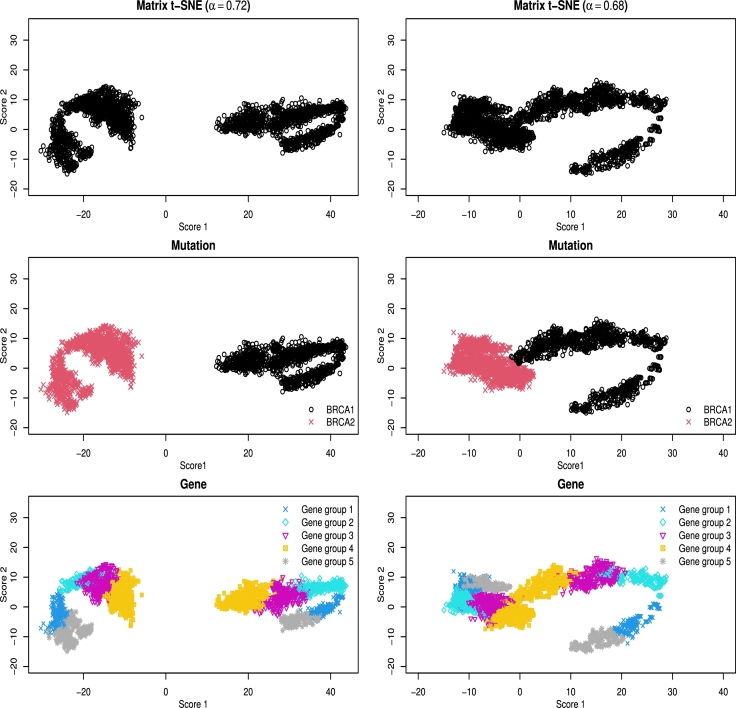
Impact of *α* selection on embeddings for the breast cancer dataset (40 × 25) using Matrix t-SNE at *α*^⁎^ = 0.72 chosen based on the *SSB*/*SST* metric and *α*^⁎^ = 0.68 chosen based on ARI and NMI metrics.

**Fig. D.18:**
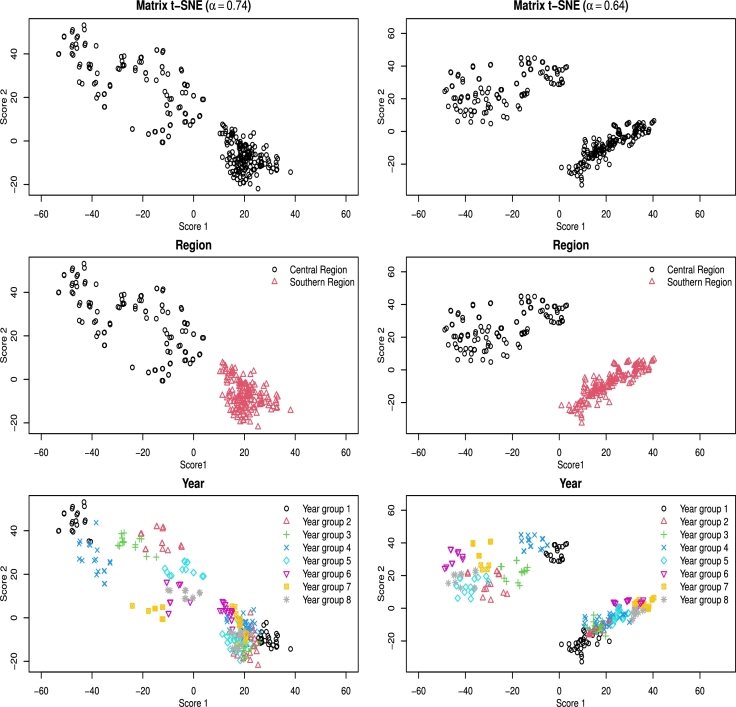
Impact of *α* selection on embeddings for the temperature dataset (5 × 55) using Matrix t-SNE at *α*^⁎^ = 0.74 chosen based on the *SSB*/*SST* metric and *α*^⁎^ = 0.64 chosen based on ARI and NMI metrics.

## Appendix A Derivation of matrix t-SNE gradient

In order to make the derivation (8), we define auxiliary variables $r_{ij}^{k},c_{kl}^{i},Z^{r}$, and $Z^{c}$ as follows

$$r_{ij}^{k}=d(Y_{ik},Y_{jk}),\;c_{kl}^{i}=d(Y_{ik},Y_{il})$$

$$Z^{r}=\sum_{i\neq j}{\left(1+\sum_{k=1}^{K}{\left(r_{ij}^{k}\right)}^{2}\right)}^{-1},\;Z^{c}=\sum_{k\neq l}{\left(1+\sum_{i=1}^{I}{\left(c_{kl}^{i}\right)}^{2}\right)}^{-1}.$$

We note that, if $Y_{ik}$ changes, the only pairwise distances $r_{ij}^{k}$ and $r_{ji}^{k}$ for all *j* and $c_{kl}^{i}$ and $c_{lk}^{i}$ for all *l* are changed. Thus, the gradient of the cost function *C* with respect to $Y_{ik}$ is given by

(A.1) $$\frac{\partial C}{\partial Y_{ik}}=\alpha \sum_{j=1}^{I}\left(\frac{\partial C}{\partial r_{ij}^{k}}\frac{\partial r_{ij}^{k}}{\partial Y_{ik}}+\frac{\partial C}{\partial r_{ji}^{k}}\frac{\partial r_{ji}^{k}}{\partial Y_{ik}}\right)+(1-\alpha )\sum_{l=1}^{K}\left(\frac{\partial C}{\partial c_{kl}^{i}}\frac{\partial c_{kl}^{i}}{\partial Y_{ik}}+\frac{\partial C}{\partial c_{lk}^{i}}\frac{\partial c_{lk}^{i}}{\partial Y_{ik}}\right)=2\alpha \sum_{j=1}^{I}\frac{\partial C}{\partial r_{ij}^{k}}(Y_{ik}-Y_{jk})\frac{1}{r_{ij}^{k}}+2(1-\alpha )\sum_{l=1}^{K}\frac{\partial C}{\partial c_{kl}^{i}}(Y_{ik}-Y_{il})\frac{1}{c_{kl}^{i}}.$$

First, the gradient $\frac{\partial C}{\partial r_{ij}^{k}}$ is computed from the Kullback-Leibler divergence (7) as follows

$$\frac{\partial C}{\partial r_{ij}^{k}}=-\alpha \sum_{i^{\prime }\neq j^{\prime }}p_{i^{\prime }j^{\prime }}^{r}\frac{\partial \log q_{i^{\prime }j^{\prime }}^{r}}{\partial r_{ij}^{k}}=-\alpha \sum_{i^{\prime }\neq j^{\prime }}p_{i^{\prime }j^{\prime }}^{r}\frac{\partial (\log q_{i^{\prime }j^{\prime }}^{r}Z^{r}-\log Z^{r})}{\partial r_{ij}^{k}}=-\alpha \sum_{i^{\prime }\neq j^{\prime }}p_{i^{\prime }j^{\prime }}^{r}\left\{\frac{1}{q_{i^{\prime }j^{\prime }}^{d}Z^{r}}\frac{\partial {\left(1+\sum_{k^{\prime }}{\left(r_{i^{\prime }j^{\prime }}^{k^{\prime }}\right)}^{2}\right)}^{-1}}{\partial r_{ij}^{k}}-\frac{1}{Z^{r}}\frac{\partial Z^{r}}{\partial r_{ij}^{k}}\right\}=-\alpha \sum_{i^{\prime }\neq j^{\prime }}p_{i^{\prime }j^{\prime }}^{r}\frac{1}{q_{i^{\prime }j^{\prime }}^{r}Z^{r}}\left\{-{\left(1+\sum_{k^{\prime }}{\left(r_{i^{\prime }j^{\prime }}^{k^{\prime }}\right)}^{2}\right)}^{-2}\right\}\frac{\partial \sum_{k^{\prime }}{\left(r_{i^{\prime }j^{\prime }}^{k^{\prime }}\right)}^{2}}{\partial r_{ij}^{k}}+\frac{\alpha }{Z^{r}}\sum_{i^{\prime }\neq j^{\prime }}p_{i^{\prime }j^{\prime }}^{r}\frac{\partial Z^{r}}{\partial r_{ij}^{k}}.$$

Here, the gradients $\frac{\partial \sum_{k^{\prime }}{\left(r_{i^{\prime }j^{\prime }}^{k^{\prime }}\right)}^{2}}{\partial r_{ij}^{k}}$ and $\frac{\partial Z^{r}}{\partial r_{ij}^{k}}$ are zero for all $i^{\prime }\neq i$, $j^{\prime }\neq j$, $k^{\prime }\neq k$ and $\sum_{i^{\prime }\neq j^{\prime }}p_{i^{\prime }j^{\prime }}^{r}=1$. Thus, the gradient $\frac{\partial C}{\partial r_{ij}^{k}}$ is given by

$$\frac{\partial C}{\partial r_{ij}^{k}}=2\alpha p_{ij}^{r}\frac{1}{q_{ij}^{r}Z^{r}}{\left(1+\sum_{k^{\prime }}{\left(r_{ij}^{k^{\prime }}\right)}^{2}\right)}^{-2}r_{ij}^{k}-2\alpha \frac{1}{Z^{r}}{\left(1+\sum_{k^{\prime }}{\left(r_{ij}^{k^{\prime }}\right)}^{2}\right)}^{-2}r_{ij}^{k}=2\alpha p_{ij}^{r}{\left(1+\sum_{k^{\prime }}{\left(r_{ij}^{k^{\prime }}\right)}^{2}\right)}^{-1}r_{ij}^{k}-2\alpha q_{ij}^{r}{\left(1+\sum_{k^{\prime }}{\left(r_{ij}^{k^{\prime }}\right)}^{2}\right)}^{-1}r_{ij}^{k}=2\alpha (p_{ij}^{r}-q_{ij}^{r}){\left(1+\sum_{k^{\prime }}{\left(r_{ij}^{k^{\prime }}\right)}^{2}\right)}^{-1}r_{ij}^{k}.$$

Substituting the above into the first term in (A.1), we obtain the gradient

$$2\alpha \sum_{j=1}^{I}\frac{\partial C}{\partial r_{ij}^{k}}(Y_{ik}-Y_{jk})\frac{1}{r_{ij}^{k}}=4\alpha^{2}\sum_{j}\left\{(p_{ij}^{r}-q_{ij}^{r}){\left(1+\sum_{k^{\prime }}{\left(r_{ij}^{k^{\prime }}\right)}^{2}\right)}^{-1}\right\}(Y_{ik}-Y_{jk})=4\alpha^{2}\sum_{j}\left\{(p_{ij}^{r}-q_{ij}^{r}){\left(1+\sum_{k^{\prime }}d{\left(Y_{ik^{\prime }},Y_{jk^{\prime }}\right)}^{2}\right)}^{-1}\right\}(Y_{ik}-Y_{jk}).$$

Similarly to the first term, we derive the second term in (A.1) as

$$2(1-\alpha )\sum_{l=1}^{K}\frac{\partial C}{\partial c_{kl}^{i}}(Y_{ik}-Y_{il})\frac{1}{c_{kl}^{i}}=4{\left(1-\alpha \right)}^{2}\sum_{l}\left\{(p_{kl}^{c}-q_{kl}^{c}){\left(1+\sum_{i^{\prime }}d{\left(Y_{i^{\prime }k},Y_{i^{\prime }l}\right)}^{2}\right)}^{-1}\right\}(Y_{ik}-Y_{il}).$$

Finally, we obtain the gradient (8) as

$$\frac{\partial C}{\partial Y_{ik}}=4\alpha^{2}\sum_{j=1}^{I}(p_{ij}^{r}-q_{ij}^{r}){\left(1+\sum_{k^{\prime }=1}^{K}d{\left(Y_{ik^{\prime }},Y_{jk^{\prime }}\right)}^{2}\right)}^{-1}(Y_{ik}-Y_{jk})+\;4{\left(1-\alpha \right)}^{2}\sum_{l=1}^{K}(p_{kl}^{c}-q_{kl}^{c}){\left(1+\sum_{i^{\prime }=1}^{I}d{\left(Y_{i^{\prime }k},Y_{i^{\prime }l}\right)}^{2}\right)}^{-1}(Y_{ik}-Y_{il}).$$

As in [7], the gradient update with a momentum term is given by

$$Y^{\left(m\right)}=Y^{\left(m-1\right)}-\eta \frac{\partial C}{\partial Y}+\gamma (m)(Y^{\left(m-1\right)}-Y^{\left(m-2\right)}),$$

where $Y^{\left(m\right)}$ is the solution at iteration *m*, *η* is the learning rate, and γ(m) is the momentum at iteration *m*.

## Appendix C Classical t-SNE with combined distance

As introduced in Section 1, the classical t-SNE with the convex combined distance (the classical t-SNE with combined distance) is an alternative for visualizing matrix-framed data. This method combines row-wise and column-wise distances into a single metric to accommodate the matrix structure. Specifically, we could consider the following combined distance:

$$d(X_{ik},X_{jl})=\alpha \cdot d(X_{i\cdot },X_{j\cdot })+(1-\alpha )\cdot d(X_{\cdot k},X_{\cdot l})$$

where $d(X_{i\cdot },X_{j\cdot })$ is the row-wise distance, $d(X_{\cdot k},X_{\cdot l})$ is the column-wise distance, and α∈[0,1] is a weight parameter to balance row-wise and column-wise distances. Using this combined distance, the conditional probabilities in the high-dimensional space are computed as:

$$p_{\left(j,l)|(i,k\right)}=\frac{\exp(-d{\left(X_{ik},X_{jl}\right)}^{2}/2\sigma_{ik}^{2})}{\sum_{\left(i^{\prime },k^{\prime })\neq (j^{\prime },l^{\prime }\right)}\exp(-d{\left(X_{i^{\prime }k^{\prime }},X_{j^{\prime }l^{\prime }}\right)}^{2}/2\sigma_{i^{\prime }k^{\prime }}^{2})},$$

where $\sigma_{ik}$ is determined by a single perplexity parameter.

While this approach incorporates the matrix structure by combining row-wise and column-wise distances, it relies on a single perplexity parameter. This reliance makes the visualization sensitive to differences in the scales of rows and columns distances, leading to an imbalance in preserving both structures. Although effective in certain scenarios, this method requires careful consideration of the data's characteristics to ensure meaningful representation.

For the classical t-SNE with combined distance, we selected the weight parameter *α* using the same rule outlined in Section 2.3. The results for this method are shown in Fig. C.11 (exergame data), Fig. C.12 (breast cancer data), and Fig. C.13 (temperature data).

The right panels in each figure present the results of the classical t-SNE with combined distance for each dataset. These results demonstrate improved separation of the row-wise (motion) structure in the exergame data and both row-wise (mutation) and column-wise (gene) structures in the gene expression data compared to the classical t-SNE with unstructured distance. However, the classical t-SNE with combined distance remains sensitive to distance scale differences. In the exergame data, the smaller scale of joint distances leads to joints forming the dominant clusters, with motions nested within them. In the gene expression data, the smaller scale of gene distances results in the dominant gene clusters. In contrast, in the temperature data, classical t-SNE with unstructured distance captures the row-wise (region) structure, but applying a convex combination of distances distorts the representation, failing to preserve both marginal structures. On the other hand, Matrix t-SNE avoids scale sensitivity by independently applying perplexities to row-wise and column-wise structures and automatically optimizing the weight through cost minimization. This removes the need for adjustments, making it robust to variations in data characteristics. As a result, Matrix t-SNE is particularly effective for complex datasets with significant scale differences or unclear distance combinations, providing consistent and reliable visualizations. These methods complement each other and can be chosen based on the data's complexity and structural properties.

## Appendix D Sensitivity analysis to the selection of *α*

In the sensitivity analysis of *α* selection, we applied a bootstrap approach (Lim et al. [28]) to assess the variability of clustering performance across different *α* values. Specifically, for the exergame and breast cancer (BRCA) datasets, we generated 30 bootstrap samples by resampling individuals or subjects with replacement. For each bootstrap sample, we computed the Adjusted Rand Index (ARI), Normalized Mutual Information (NMI), and F-statistics (SSB/SST ratio) across all *α* values from 0.1 to 0.9. This procedure allowed us to evaluate the stability of *α* selection based on different clustering performance measures while accounting for sample variability. The results are displayed in Fig. D.14 (exergame data) and Fig. D.15 (BRCA data). These figures show that clustering performance remains stable across different measures and bootstrapped datasets, indicating that *α* selections change little across all three measures. The temperature dataset was excluded from this analysis due to its small sample size (five cities) and the nature of the data structure, which makes bootstrap resampling inappropriate in this context.

In addition to evaluating clustering performance, we further investigated how the embedded maps change with different selections of *α*. For each dataset, we generated two maps of Matrix t-SNE: one using the *α* value selected by the F-statistic and the other using the *α* value commonly selected by ARI and NMI. The resulting maps are shown in Fig. D.16 (exergame data), Fig. D.17 (BRCA data), and Fig. D.18 (temperature data). The comparison of these maps indicates that the embedded structures do not change dramatically depending on the selected *α*, likely because the *α* values selected by the different measures were relatively close in this analysis.
